# PIK3CA Gene Mutations and Overexpression: Implications for Prognostic Biomarker and Therapeutic Target in Chinese Esophageal Squamous Cell Carcinoma

**DOI:** 10.1371/journal.pone.0103021

**Published:** 2014-07-23

**Authors:** Lin Wang, Ling Shan, Shaokai Zhang, Jianming Ying, Liyan Xue, Yanling Yuan, Yongqiang Xie, Ning Lu

**Affiliations:** 1 Department of Pathology, Cancer Hospital, Peking Union Medical College, Chinese Academy of Medical Sciences, Beijing, P. R. China; 2 Department of Epidemiology, Cancer Hospital, Peking Union Medical College, Chinese Academy of Medical Sciences, Beijing, P. R. China; Sapporo Medical University, Japan

## Abstract

**Aims:**

To evaluate PIK3CA gene mutations and PIK3CA expression status in Chinese esophageal squamous cell carcinoma (ESCC) patients, and their correlation with clinicopathological characteristics and clinical outcomes.

**Methods:**

Direct sequencing was applied to investigate mutations in exons 9 and 20 of PIK3CA in 406 Chinese ESCC patients. PIK3CA expression was evaluated using immunohistochemistry analysis. The associations of PIK3CA gene mutations and PIK3CA expression with clinicopathological characteristics and clinical outcome were examined.

**Results:**

Thirty somatic point mutations (30/406, 7.4%) were identified in exon 9 whereas no mutations were detected in exon 20. PIK3CA mutations were not correlated with clinicopathological characteristics or clinical outcomes. However in the ESCC patients with family cancer history, PIK3CA mutations were independently correlated with worse overall survival (multivariate hazard ratio (HR) = 10.493, 95% CI: 2.432–45.267, P = 0.002). Compared to normal esophageal tissue, PIK3CA was significantly overexpressed in cancer tissue (P<0.001). PIK3CA overexpression was independently associated with higher risk of local recurrence (multivariate HR  = 1.435, 95% CI: 1.040–1.979, P = 0.028). In female ESCC patients, PIK3CA overexpression was independently correlated with worse overall survival (multivariate HR  = 2.341, 95% CI: 1.073–5.108, P = 0.033).

**Conclusions:**

Our results suggest PIK3CA gene mutation and overexpression could act as biomarkers for individualized molecular targeted therapy for Chinese ESCC patients.

## Introduction

Esophageal squamous cell carcinoma (ESCC) is the predominant histological subtype of esophageal cancer in East Asian countries [1], accounting for more than 90% of the total esophageal cancer cases [1]. Epidemiologic studies have reported that alcohol and tobacco use as well as low consumption of fruits and vegetables are risk factors of ESCC worldwide [2]. In some regions of north and central China, the incidence of ESCC exceeds 100/100,000 cases per year [3]. Despite the development of various therapeutic strategies for ESCC, including surgery, chemotherapy, radiotherapy, and combination therapy, the prognosis for ESCC patients remains unfavorable. The 5-year survival rates of ESCC were between 11.1% and 56.5% depending on the clinical stage at the time of diagnosis [4]. Therefore new therapeutic targets for ESCC treatment are urgently needed.

With the development of high-throughput genome sequencing and screening technologies, more and more cancer-associated genes have been identified to serve as potential therapeutic targets or prognostic indicators [5]. Using whole-genome and whole-exome sequencing analysis, PIK3CA has been identified as a significantly mutated gene in 4.5% of 88 ESCC cases [6]. In a study using high-throughput genotyping analysis in 80 ESCC cases, PIK3CA was revealed with the highest mutation frequency (11.5%) [7]. In the studies specifically examining PIK3CA mutations, the frequency of PIK3CA mutation were detected in a range from 2.2% to 21% in ESCC patients [7]–[12]. Given the frequency of the mutation, PIK3CA is at the forefront of investigations in ESCC to serve as a potential therapeutic target.

PIK3CA gene is located on 3q26.3 chromosome and encodes the catalytic p110 alpha subunit of phosphoinositide 3-kinase (PI3K). The PI3K signaling pathway is deregulated in many types of cancer [13] and only PIK3CA has been reported to be mutated and amplified [14], [15]. More than 80% of the PIK3CA mutations detected were localized in exons 9 and 20 (helical and kinase domain) [16]–[18], with three ‘hot-spot’ mutations, E542K, E545K and H1047R. Cancer cells with PIK3CA mutations have been demonstrated to acquire enhanced sensitivity to PI3K pathway inhibitors [12], [19]–[22]. Moreover clinical phase I trials revealed that the PIK3CA mutation is associated with response to PI3K pathway inhibitors [23]–[25].

In this study, we sought to determine the frequency of PIK3CA mutation and PIK3CA expression status in 406 Chinese ESCC patients. The associations of PIK3CA mutations and PIK3CA expression with clinicopathological factors and patient outcome were evaluated. To the best of our knowledge, this is, by far, the largest study on the prognostic role of PIK3CA mutations and PIK3CA expressions in ESCC to date.

## Materials and Methods

### Patients and samples

This study included 406 ESCC patients, who underwent radically resection at Cancer Hospital, Chinese Academy of Medical Sciences from 2004 to 2007. The study protocol was approved by the ethics review board of the Chinese Academy of Medical Sciences. We have obtained written informed consent from all study participants under the approval of the ethics review board of the Chinese Academy of Medical Sciences. All of the procedures were done in accordance with the Declaration of Helsinki and relevant policies in China. All the patients did not receive treatments (chemotherapy, radiotherapy or immunotherapy) prior to surgery. Clinicopathological information was available for all the patients, including age, gender, tobacco use, alcohol use, family cancer history, tumor location, pathologic stage, differentiation, lymph node metastasis, tumor embolus, local recurrence and prognosis (**Table 1**). Local recurrence in the esophageal cancer was defined as the recurrence at the site of the anastomosis and/or local region, including mediastinal lymph nodes and supraclavicular lymph nodes [26].

**Table 1:** PIK3CA gene mutations, PIK3CA expression and clinicopathological characteristics in the ESCC patients.

Clinical,epidemiological or pathological feature	Total	PIK3CA		P value	PIK3CA		P value
	(N)	Mutant (%)	Wild-type (%)		Positive(+)(%)	Negative(-)(%)	
**All cases**	406	30(7.4)	376(92.6)		250(61.6)	156(38.4)	
**Age**							
** Median age(years)**		59	59		59	59	
** <59 years**	195	13(6.7)	182(93.3)	0.593	119(61.0)	76(39.0)	0.826
** ≥59 years**	211	17(8.1)	194(91.9)		131(62.1)	80(37.9)	
**Sex**							
** Male**	318	26(8.2)	292(91.8)	0.249	200(62.9)	118(37.1)	0.300
** Female**	88	4(4.6)	84(95.4)		50(56.8)	38(43.2)	
**Tobacco use**							
** Yes**	255	23(9.0)	232(90.9)	0.103	157(61.6)	98(38.4)	0.997
** No**	151	7(4.6)	144(95.4)		93(61.6)	58(38.4)	
**Alcohol use**							
** Yes**	222	19(8.6)	203(91.4)	0.322	145(65.3)	77(34.7)	0.089
** No**	184	11(6.0)	173(94.0)		105(57.0)	79(42.9)	
**Family cancer history**							
** Yes**	85	5(5.9)	80(94.1)	0.550	46(54.1)	39(45.9)	0.112
** No**	321	25(7.8)	296(92.2)		204(63.6)	117(36.4)	
**Tumor location**							
** Upper thoracic**	38	2(5.2)	36(94.8)	0.467^c^	25(65.8)	13(34.2)	0.425
** Middle thoracic**	245	16(6.5)	229(93.5)		155(63.2)	90(36.8)	
** Lower thoracic**	123	12(9.8)	111(90.2)		70(56.9)	53(43.1)	
**Pathologic T classification**							
** T1**	99	8(8.0)	91(92.0)	0.607	54(54.6)	45(45.4)	0.075
** T2**	142	8(5.6)	134(94.4)		84(59.2)	58(40.8)	
** T3**	165	14(8.5)	151(91.5)		112(67.9)	53(32.1)	
**Degree of differentiation**							
** Well**	72	5(7.0)	67(93.0)	0.512	41(56.9)	31(43.1)	0.595
** Moderate**	246	16(6.5)	230(93.5)		152(61.8)	94(38.2)	
** Poor**	88	9(10.2)	79(89.8)		57(64.8)	31(35.2)	
**Lymph node metastasis**							
** Yes**	176	13(7.4)	163(92.6)	0.998	112(63.6)	64(36.4)	0.455
** No**	230	17(7.4)	213(92.6)		138(60.0)	92(40.0)	
**Tumor embolus**							
** Yes**	54	8(14.8)	46(85.2)	0.050^c^	37(68.5)	17(31.5)	0.260
** No**	352	22(6.3)	330(93.7)		213(60.5)	139(39.5)	
**Local recurrence**							
** Yes**	181	13(7.2)	168(92.8)	0.886	124(68.5)	57(31.5)	0.010^a^
** No**	225	17(7.6)	208(92.4)		126(56.0)	99(44.0)	
**Prognosis**							
** Dead**	226	19(8.4)	207(91.6)	0.380	149(65.9)	77(34.1)	0.043^a^
** Survival**	180	11(6.1)	169(93. 9)		101(56.1)	79(43.9)	

a The P-value is significant.

c Fisher's exact probability test.

### PCR and direct sequencing of PIK3CA exon 9 and exon 20

Genomic DNA was extracted from 406 formalin-fixed and paraffin-embedded (FFPE) ESCC tissue samples with minimum of 75% malignant cells. DNA was extracted using the QIAamp DNA Mini kit (Qiagen, Hilgen, Germany) according to manufacturer's instructions. For those samples with PIK3CA mutations, DNA from the paired normal tissue samples was extracted. The PCR amplifications targeting exon 9 and 20 of the PIK3CA gene were performed. For exon 9 amplification, two pairs of primers were used. The first pair included PIK3CA 9F-1, 5′-GTATTTGCTTTTTCTGTAAATCATCTG-3′ and PIK3CA 9R-1, 5′-CATGCTGAGATCAGCCAAATTC-3′. For the samples, which were considered to have exon 9 mutations, the second primer pair was applied. The second primer pair was as follows: PIK3CA 9F-2, 5′-AGTAACAGACTAGCTAGAGAC-3′ and PIK3CA 9R-2, 5′-AAAATCATGTAAATTCTGCTTTATT-3′. For the exon 20, the following primers were used: PIK3CA 20F, 5′-CTCTGGAATGCCAGAACTAC-3′ and PIK3CA 20R, 5′-ATGCTGTTTAATTGTGTGGAAG-3′. The PCR mixture contained 50 ng of DNA, 5 pmol/L of each primer, 2.5 nmol/L of each dNTP, and 1.25 U of Taq Gold DNA polymerase in 11.5 µL of buffer containing 0.04 µmol/L of Mg^2+^. The cycling conditions were as follows: initial denaturation at 95°C for 10 min, followed by 38 cycles of 95°C for 30 s, 56°C for 30 s, 72°C for 45 s, and a single extension step at 72°C for 10 min. PCR products were treated with Exonuclease I/Antarctic Phosphatase (New England Biolabs, MA, USA) at 37°C for 30 min and 80°C for 15 min. The sequencing primers were the same with the PCR primers. Purified sequencing PCR products were sequenced on an ABI 3500XL analyzer (Applied Biosystems, Caarlsbad, CA) using POP7 polymer.

### ESCC Tissue Microarray (TMA)

All samples were reviewed to confirm for ESCC by a pathologist (W.L.). Four hundred and six FFPE ESCC samples, of which two hundred and twenty-six samples had paired normal tissue samples, were constructed onto 10 TMAs. For the patients at pT2 or pT3 stage, two tumor cores and one normal core of 1.0 mm diameter were taken based on hematoxylin and eosin (H&E)-stained sections of each sample. For the patients at pT1 stage, two tumor cores and one normal core of 0.6 mm diameter were taken and constructed onto TMAs. The collection of these specimens was approved by the local ethics committee.

### Immunohistochemistry assay

Immunohistochemical staining was performed on 4 µm-thick TMAs and was carried out by a pathologist (W.L.). Briefly, the slides were deparaffinized and antigen retrieval was then performed in a steam cooker for 1.5 minutes in 1 mM citric acid, pH 6.0 (Maixin Biological Techology Co. Ltd., Fuzhou, China). PIK3CA (p110α) rabbit anti-human monoclonal antibody (Cell Signaling Technology, USA) was applied at 1∶100 dilution for 1 h. Universal secondary antibody (DAKO) was applied for 15 min. Diaminobenzidine or 3-amino-9-ethylcarbazole was used as chromogens and slides were counterstained with haematoxylin before mounting.

Positive staining for PIK3CA was demonstrated by the presence of brown-yellow or brown granules. PIK3CA expression was scored on a scale from grade 0 to 3+: 0, no staining; 1+, <50% with weak or strong intensity; 2+, ≥50% with weak intensity; and 3+, ≥50% with strong intensity. For statistical analysis, we classified PIK3CA expression of score 0 or 1+ as negative and score 2+ or 3+ as positive [11]. Immunohistochemical staining was independently scored by 2 pathologists (X.L.Y. and Y.J.M.) without any clinicopathological information.

### Statistical analysis

Statistical comparisons were performed using the SPSS 16.0 software package. The association between PIK3CA mutations or PIK3CA expression and the clinicopathological variables were performed using the χ^2^-test or Fisher's exact probability test. The correlation between PIK3CA mutation and PIK3CA expression was determined using the Spearman's test with determination of correlation coefficients(r) and associated probabilities (P). All P-values were two-tailed and a P-value of <0.05 was considered significant. Univariate analysis and multivariate analysis were performed using the Cox proportional hazard regression analysis. Estimation of overall survival was calculated using the Kaplan-Meier method and the statistical differences were analyzed using the log-rank test.

## Results

### Clinicopathological characteristics of the ESCC samples

A total of 406 ESCC cases, consisted of 318 men (78.3%) and 88 (21.7%) women, were included in this study. The patients' clinicopathological characteristics are summarized in **Table 1**. Their median age was 59 years (range 35 to 80 years). Of all patients, there were 255 cases (62.8%) with smoking habit and 222 cases (54.7%) with drinking habit. In terms of tumor location, the majority of the patients had middle (60.3%) or lower thoracic (30.3%) ESCC. The tumors were poorly differentiated in 21.7%, moderately differentiated in 60.6%, and well-differentiated in 17.7%. Tumor status at time of resection was pT3 disease in 165 (40.6%); pT1 and pT2 were in 99 (24.4%) and 142 (35.0%) of the patients, respectively. There were 43.3% of the cases with lymph node metastasis and 1.3% of the cases with tumor embolus.

Follow-up data were complete for the 406 ESCC cases. Median follow-up was 62 months (range 4–107). The local recurrence was occurred in 44.6% of the cases and estimated 5-year overall survival (OS) was 45.3%.

### PIK3CA mutations and patients characteristics

**Figure 1:**
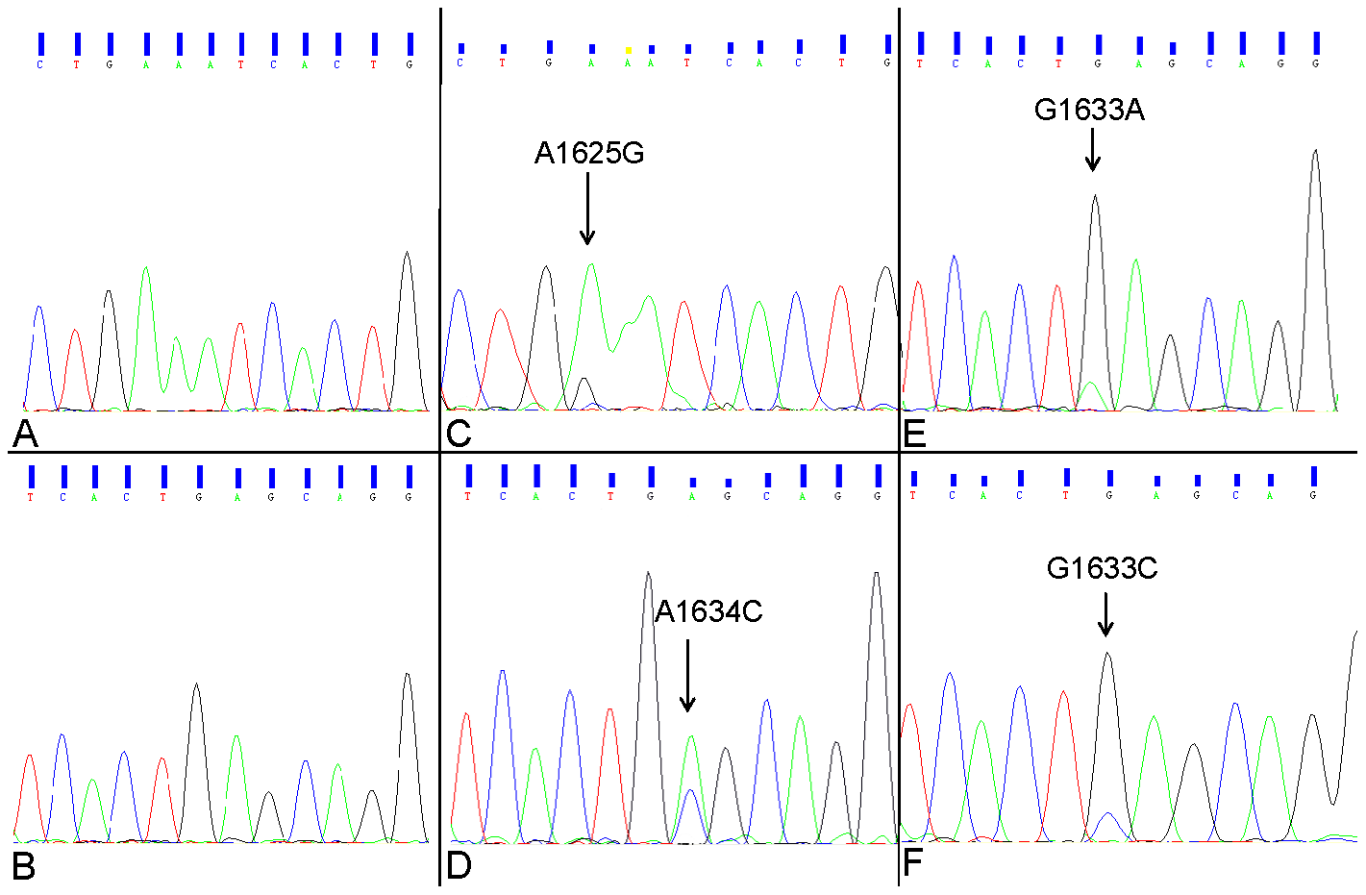
Mutational analysis of PIK3CA exons 9 and 20 in ESCC. Representative chromatograms of the four mutations detected in exon 9 of the PIK3CA gene and the corresponding wild-type region. (A) and (B) Wild-type sequence. (C) Mutant sequence E542Q (A1625G); (D) Mutant sequence E545A (A1634C); (E) Mutant sequence E545Q (G1633A); (F) Mutant sequence E545K (G1633C). The arrows indicate the location of the somatic mutations.

We conducted mutational analysis to examine PIK3CA exons 9 and 20 mutations in 406 ESCC cases. PIK3CA mutations were detected in 30 (7.4%) ESCC samples and no mutations were identified in correspondent normal tissues (**Table 1** **,** **Figure 1**). The mutations were only observed in exon 9. The most common mutation was the c.1634A>C (p.E545A) mutation, which occurred in 24 samples. Other mutations including c.1625A>G (p.E542G) mutation, c.1633G>C (p.E545K) and c.1633G>A (p.E545Q) were detected in 3, 2 and 1 samples, respectively (**Table S1**).

The association of PIK3CA mutations with clinicopathological features and clinical outcome were statistically assessed (**Table 1**). PIK3CA mutations were not correlated with clinicopathological characteristics, including age, gender, tobacco use, alcohol use, family cancer history, tumor location, pathologic stage, differentiation, lymph node metastasis, tumor embolus, local recurrence, and prognosis. However for the patients with the age <59 years or with family cancer history, PIK3CA mutations were correlated with worse overall survival (log rank P = 0.039, 0.026 respectively) (**Figure S1**). In the multivariate Cox regression analysis, PIK3CA mutations were found to be independently associated with worse overall survival in the patients with family cancer history (multivariate HR = 10.493, 95% CI: 2.432–45.267, P = 0.002) (**Table 2**).

**Table 2:** Multivariate Cox regression analysis of clinical variables on overall survival accord to (A) PIK3CA mutation status and (B) PIK3CA expression status.

Variable	N	Multivariate analysis		
		Hazard ratio	95% CI	P value
**(A) PIK3CA mutation status**				
** All ESCC patients** a	406	1.256	0.748–2.108	0.388
** ESCC patients with age<59 years** b	195	1.363	0.753–2.467	0.307
** ESCC patients with family history of cancer** c	85	10.493	2.432–45.267	0.002
**(B) PIK3CA expression status**				
** All ESCC patients** a	406	1.072	0.794–1.447	0.651
** ESCC patients with age≥59 years** b	211	1.237	0.753–2.035	0.401
** Female ESCC patients** d	88	2.341	1.073–5.108	0.033
** Non-smoking ESCC patients** e	151	1.144	0.665–1.968	0.627

a Adjust by age, gender, tobacco use, alcohol use, family cancer history, tumor location, pathologic T classification, degree of differentiation, lymph node metastasis, tumor embolus and local recurrence.

b Adjust by gender, tobacco use, alcohol use, family cancer history, tumor location, pathologic T classification, degree of differentiation, lymph node metastasis, tumor embolus and local recurrence.

c Adjust by age, gender, tobacco use, alcohol use, tumor location, pathologic T classification, degree of differentiation, lymph node metastasis, tumor embolus and local recurrence.

d Adjust by age, tobacco use, alcohol use, family cancer history, tumor location, pathologic T classification, degree of differentiation, lymph node metastasis, tumor embolus and local recurrence.

e Adjust by age, gender, alcohol use, family cancer history, tumor location, pathologic T classification, degree of differentiation, lymph node metastasis, tumor embolus and local recurrence.

### PIK3CA expression and patients characteristics

**Figure 2:**
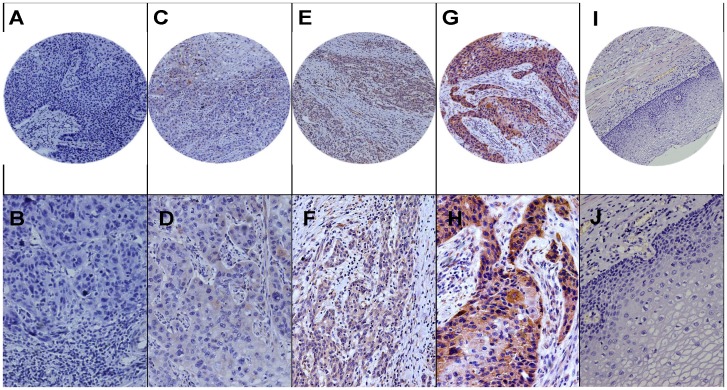
PIK3CA expression in ESCC. Representative immunohistochemistry images of PIK3CA expression across multiple tumor grades. PIK3CA protein was localized on the cytoplasm of tumor cells. Tumors were scored on a scale from grade 0 to 3+. (A) grade 0, 100×; (B) grade 0, 200×; (C) grade 1+, 100×; (D) grade 1+, 200×; (E) grade 2+, 100×; (F) grade 2+, 200×; (G) grade 3+, 100×; (H) grade 3+, 200×; (I) normal mucosa, 100×; and (J) normal mucosa, 200×.

The PIK3CA expression status was examined in 406 ESCC tumor tissues and 223 paired normal tissues. The PIK3CA protein was mainly localized in cytoplasm. The expression of PIK3CA was detected in 41 of 223 (18.4%) normal tissue samples and 250 of 406 (61.6%) tumor samples (**Figure 2**). The PIK3CA was significantly overexpressed in ESCC tumor specimens compared to normal samples (P<0.001) (**Table S2**).

We identified a significant correlation between PIK3CA expression and local recurrence and prognosis (P = 0.010, 0.043, respectively) (**Table 1**). In the univariate Cox regression analysis, PIK3CA overexpression was significantly associated with local recurrence (HR = 1.500, 95% CI: 1.092–2.063, P = 0.012) but not with overall survival. In the multivariate Cox model adjusted for the clinicopathlogical and epidemiological features, PIK3CA overexpression were identified to be associated with local recurrence (HR = 1.435, 95% CI: 1.040–1.979, P = 0.028). Other features, including pT stage, lymph node metastasis and tumor embolus, were significantly associated with local recurrence in the multivariate analysis (**Table 3**).

**Table 3:** Cox regression analysis for Local recurrence factors with ESCC patients.

Variables(N)	Univariate analysis		Multivariate analysis	
	Hazard ratio (95% CI)	P value	Hazard ratio (95% CI)	P value
**Pathologic T classification**		<0.001^a^		<0.001^a^
** T1(99)**	1.000			
** T2(142)**	2.273(1.393–3.707)	0.001^a^	1.868(1.139–3.064)	0.013^a^
** T3(165)**	4.260(2.673–6.791)	<0.001^a^	3.135(1.936–5.078)	<0.001^a^
**Degree of differentiation**		0.024^a^		0.280
** Well(72)**	1.000			
** Moderate(246)**	1.348(0.872–2.085)	0.179	1.257(0.805–1.962)	0.315
** Poor(88)**	1.918(1.177–3.123)	0.009	1.509(0.906–2.514)	0.114
**Lymph node metastasis**				
** No(230)**	1.000			
** Yes(176)**	2.532(1.878–3.412)	<0.001^a^	1.647(1.185–2.290)	0.003^a^
**Tumor embolus**				
** No(352)**	1.000			
** Yes(54)**	2.566(1.769–3.722)	<0.001^a^	1.901(1.280–2.822)	0.001^a^
**PIK3CA expression**				
** Negative(156)**	1.000			
** Positive(250)**	1.500(1.092–2.063)	0.012^a^	1.435(1.040–1.979)	0.028^a^

a The P-value is significant.

**Figure 3:**
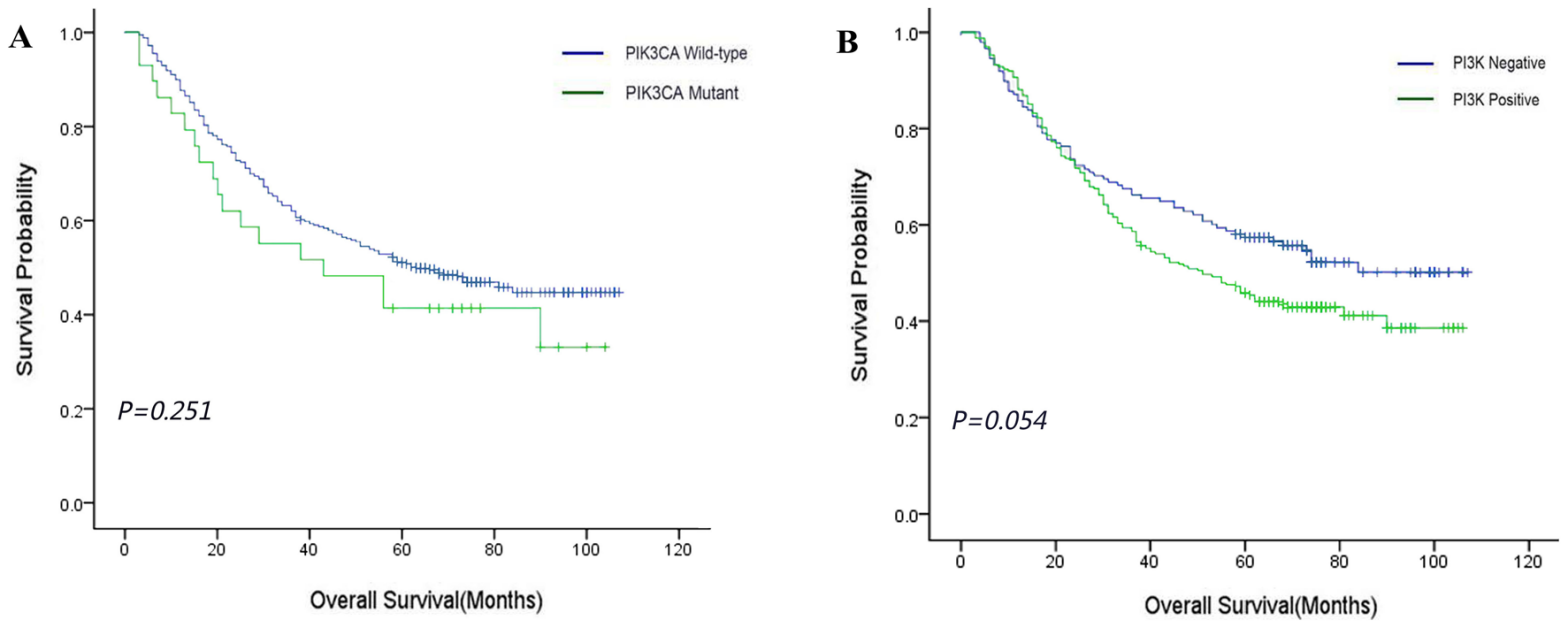
Kaplan-Meier curves for overall survival in ESCC. (A) Overall survival according to PIK3CA mutation status (P = 0.251). (B) Overall survival according to PIK3CA expression status (P = 0.054).

According to the Kaplan-Meier analysis shown in **Figure 3**, the ESCC patients with PIK3CA overexpression showed a trend towards unfavorable overall survival (P = 0.054). In the subgroups of the patients, including the patients with the age ≥59 years or without smoking habit or the female patients, PIK3CA overexpression was correlated with the overall survival (P = 0.034, 0.047, 0.025, respectively) (**Figure S2**). The multivariate Cox regression analysis revealed that in female ESCC patients PIK3CA overexpression was an independent factor associated with an increased risk for shorter overall survival (HR = 2.341, 95% CI: 1.073–5.108, P = 0.033) (**Table 2**).

### PIK3CA mutations and PIK3CA expression in ESCC

The association of PIK3CA mutation and PIK3CA expression were statistically analyzed. No significant correlation between PIK3CA mutation and PIK3CA expression was identified (r = 0.049, P = 0.326) (**Table S3**).

## Discussion

PIK3CA is an oncogene in various cancers [27], and thus it is one of the most genetically mutated genes in human cancers, including colorectal, brain and gastric cancers [27]. Also, PIK3CA mutation could constitutively active PI3K/Akt signaling cascade, leading to cell survival, proliferation, anti-apoptosis, tumorigenesis, angiogenesis, invasion and metastasis [27]–[29]. The functional region and hotspot mutation region of PIK3CA is shown in **Figure S3**. The schematic representation of mutation landscape in PIK3CA is shown in **Figure S4**. The mutation of PIK3CA has been reported in 2.2% to 21% of ESCC patients [7]–[12]. In our study, 7.4% of ESCC cases were identified with PIK3CA mutation. The discrepancy of the mutation rates in different studies is mainly caused by the detection method applied. Using direct sequencing analysis, the frequency of the PIK3CA mutation was identified in 2.2% to 7.7% of ESCC patients [9], [11], [12]. In contrast, the studies using pyrosequencing analysis or other high-sensitive mutation detection methods, the frequency of the PIK3CA mutation was observed in 11.5% to 21% of the cases [7], [8], [10]. The underlying mechanism is most probably the heterogeneous PIK3CA mutation in cancer cells. Although the direct sequencing is not a quantitative analysis method, the peak heights of the mutant and wild-type alleles could partly reflect the ratio of two types of the cells. As shown in the sequencing result in **Figure 1**, although more than 75% of the examined cells in each case were malignant cells, the peak height of the mutant alleles in most of the samples was lower than 1/3 of peak height of the wild-type alleles. It indicated that the PIK3CA mutation only occurred in relatively small amount of ESCC cancer cells. Using high-sensitive detection methods, even very low percentage of the cancer cells carrying PIK3CA mutation could be identified. Therefore PIK3CA mutation was detected with higher frequency in such studies. It suggested that if PIK3CA mutation becomes a therapeutic target in clinic in the future, the direct sequencing might not be an ideal detection methodology to be applied. The high-sensitive detection method, such as pyrosequencing, is recommended.

PIK3CA mutations were only found in exon 9 in this study, which is consistent with three other studies [9],[11],[12]. It indicated that mutations are more common in exon 9 in ESCC. However, researchers also found PIK3CA mutations in exon 20 (H1047R and H1047L) except in exon 9 [8], [30], [31]. A study reported PIK3CA mutations in exon 4 (L339F), which was rare in other reports [32]. In 30 mutations identified, majority of the mutations were c.1634A>C (p.E545A), which was in line with a study in ESCC [11]. However a few studies have reported to consider c.1634A>C as a result of the amplification of the pseudogene CES located on chromosome 22q11.2, herein not a mutation. This assumption was raised based on the identification of the c.1634A>C in both cancer and normal cells [9], [33], [34]. In our study, we did observe the c.1634A>C in almost half of the ESCC cases using the first pair of the PCR primer. However after using the second pair of PCR primers to re-examine the cases with potential mutation c.1634A>C, only 24 cases were confirmed and the mutation was not detected in the paired adjacent normal tissue. We assumed that the studies, which excluded the possibility of the c.1634A>C to be a mutation, applied unspecific PCR primers which could not prevent the intervention of the pseudogene in the sequencing analysis. Therefore the identification of c.1634A>C in both cancer and normal cells hindered the authors to consider the fact that the c.1634A>C mutation did exist.

The exon 9 of PIK3CA encodes the helical domain of the p110α subunit. The mutations in codon 542 or 545 could relieve the inhibitory effect of p85 on p110α leading to increased PI3K activity and enhanced downstream signaling elements, including AKT [35], [36]. Over ten different PI3K inhibitors have been tested in various cancer cells, i.e. breast cancer, lung cancer, colorectal cancer cells, etc. Cells with PIK3CA mutation have been demonstrated to be more susceptible to the inhibitors than those without [12], [19]–[22]. The PI3K inhibitor LY294002 has been shown to reduce proliferation more effectively in an ESCC cell line with E545K mutation compared to the cell lines with wild type PIK3CA [12]. Currently early-phase and phase I clinical trials have proved higher response rate for the cancer patients with PIK3CA mutations treated with PI3K/AKT/mTOR pathway inhibitors than for those without the mutations [23]–[25]. However no clinical trials have been performed in ESCC. Since the mutation c.1634A>C (p.E545A) is uncommon in other cancer types, the susceptibility of the ESCC patients with c.1634A>C to the inhibitors need to be studied.

PIK3CA mutation has been reported to indicate a favorable prognosis in Japanese ESCC patients [10]. However in our study PIK3CA mutation was not identified to be associated with clinicopathological features and patient outcomes, which is consistent with two other studies in Korea and China [7], [9]. The possible reasons for the different results might due to different patient cohorts, sample sizes or ethics. Although PIK3CA mutation was not correlated with patient outcome in this cohort, it was independently associated with worse overall survival in the ESCC patients with family cancer history. It has been reported that family cancer history increased the risk of esophageal cancer [37], [38], which indicated that some persons with family cancer history might have germline genetic alterations to make them prone to ESCC. Currently we could not know clearly which signal pathway(s) might be affected in ESCC patients with family cancer history. However according to our study, the dysregulation of PI3K pathway caused by PIK3CA mutation could possibly cooperate with the altered signal pathway(s) to make the patients with family cancer history to have worse survival. As there were only 85 ESCC patients with family cancer history in this study, further study with larger sample size is required.

The overexpression of PIK3CA has been reported in various types of cancer including ESCC [11], [39]–[41]. In our study, we detected the overexpression of PIK3CA in 61.6% of ESCC patients. The PIK3CA mutation rate was dramatically less than the protein expression rate and no correlation between the mutation and the expression was identified. It indicated that the PIK3CA mutations were not the sole cause of the high expression of PIK3CA protein in ESCC samples. Other mechanisms, including the gene amplification and/or promoter hypomethylation, might contribute to the high expression of PIK3CA protein as well [9]. The association of PIK3CA overexpression with lymph node metastasis has been identified in colorectal cancer, gastric cancer, ESCC, etc. [11], [39], [42]. In this study we demonstrated an independent correlation of PIK3CA overexpression with local recurrence. It implied that PIK3CA overexpression might be able to increase catalytic activity of PI3K and subsequently overactivated PI3K/AKT pathway to promote tumor progression and metastasis in ESCC [43], [44]. In female ESCC patients specifically, PIK3CA overexpression was independently associated with shorter overall survival. The interaction of PI3K/AKT and estrogen signaling pathways in ESCC has not been well documented. In contrast, more extensive studies have been performed in breast cancer, as both PI3K and estrogen pathways play a role in the initiation and progression of breast cancer [45]–[47]. It has been demonstrated that the PI3K utilizes AKT-dependent and AKT-independent pathway in activating estrogen receptor α (ERα) in breast cancer cells and it could help the cancer cells to escape from tamoxifen-induced apoptosis [48]. Further study is required to clarify the relationship of PI3K and estrogen pathway activation in ESCC.

In conclusion, PIK3CA was mutated in 7.4% of Chinese ESCC patients and c.1634A>C (p.E545A) was the dominant mutation type. The family cancer history is a risk factor for shorter overall survival for the patients with PIK3CA mutations. PIK3CA protein overexpression was independently correlated with local recurrence and the sex was independently correlated with overall survival for the patients with PIK3CA overexpression. PI3K pathway is a potential therapeutic target in Chinese ESCC patients.

## Supporting Information

Figure S1 **Kaplan-Meier curves for overall survival in ESCC patients according to PIK3CA mutation status.** (A) Overall survival in ESCC patients with age <59 years (P = 0.039). (B) Overall survival in ESCC patients with family history of cancer (P = 0.026).
(TIF)

Figure S2 **Kaplan-Meier curves for overall survival in ESCC patients according to PIK3CA expression status.** (A) Overall survival in ESCC patients with age ≥59 years (P = 0.034). (B) Overall survival in non-smoking ESCC patients (P = 0.047). (C) Overall survival in female ESCC patients (P = 0.025).
(TIF)

Figure S3 **Functional region and hotspot mutation region of PIK3CA.**
(TIF)

Figure S4 (A) Schematic representation of mutation landscape in PIK3CA as reported in COSMIC database (assessed on May 27, 2014). (B) The overall distribution of known somatic mutations (top panel) and the respective distribution (bottom panel) in tabulated form are provided for cross-reference to somatic landscape in PIK3CA.
(TIF)

Table S1 **PIK3CA mutations in ESCC.**
(DOC)

Table S2 **PIK3CA expression in ESCC and normal tissues.**
(DOC)

Table S3 **PIK3CA expression and PIK3CA mutations in the ESCC patients.**
(DOC)
