# Supplementary figures and images for: PIK3CA Gene Mutations and Overexpression: Implications for Prognostic Biomarker and Therapeutic Target in Chinese Esophageal Squamous Cell Carcinoma
Source: PLoS One. 2014 Jul 23;9(7):e103021. doi: 10.1371/journal.pone.0103021 (PMC4108430; doi:10.1371/journal.pone.0103021)

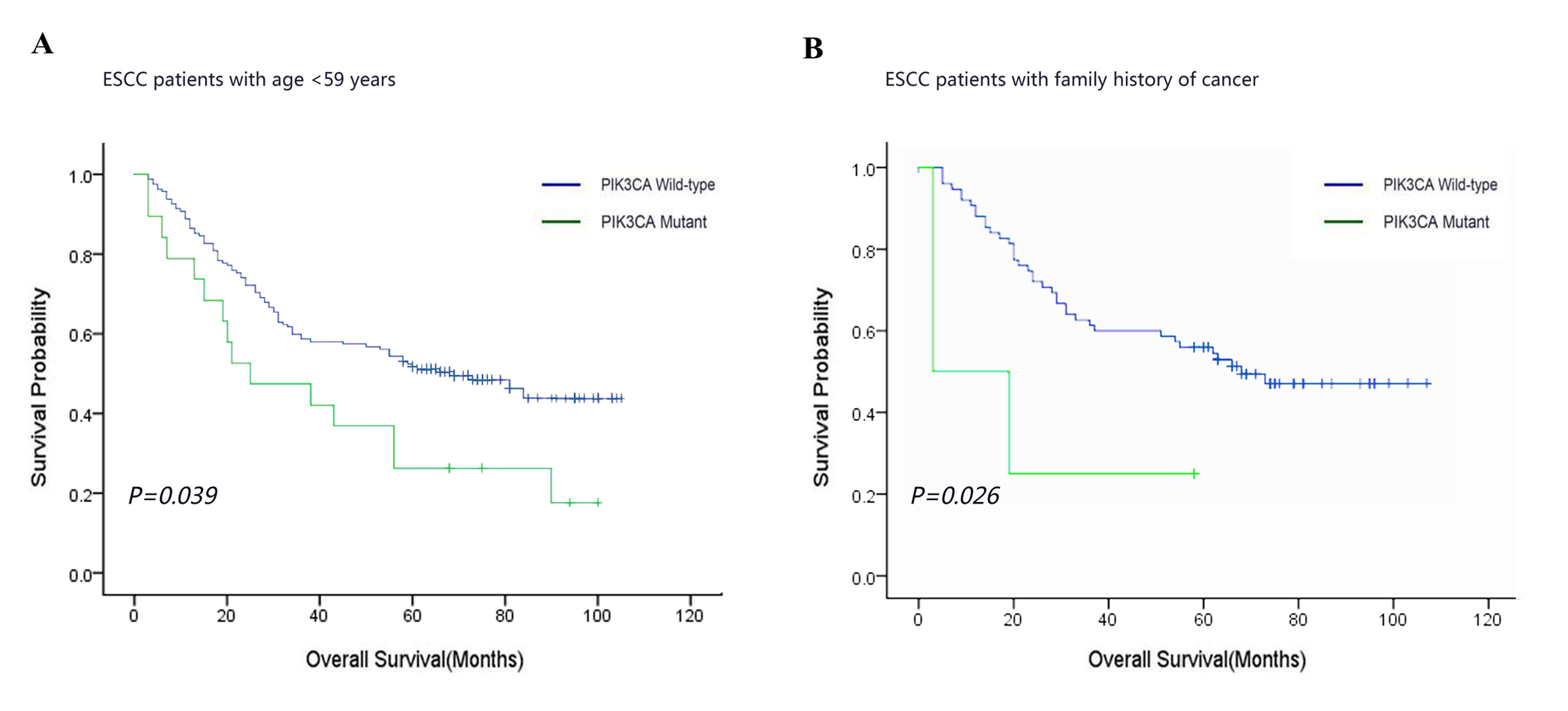

Supplement: Figure S1 — Kaplan-Meier curves for overall survival in ESCC patients according to PIK3CA mutation status. (A) Overall survival in ESCC patients with age <59 years (P = 0.039). (B) Overall survival in ESCC patients with family history of cancer (P = 0.026). (TIF) [file pone.0103021.s001.tif]

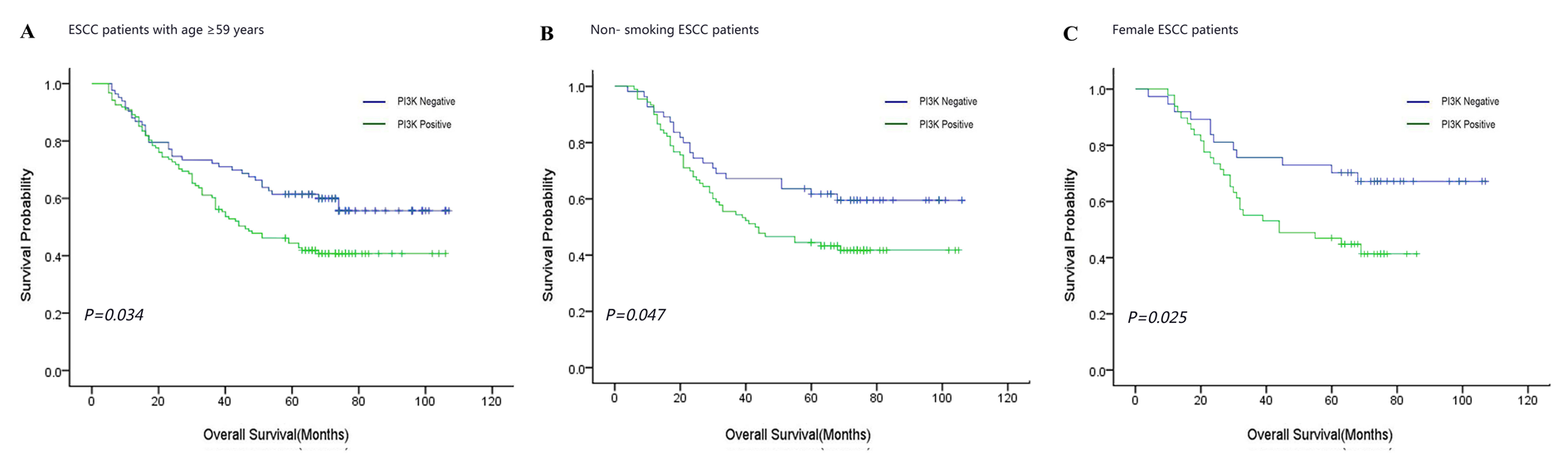

Supplement: Figure S2 — Kaplan-Meier curves for overall survival in ESCC patients according to PIK3CA expression status. (A) Overall survival in ESCC patients with age ≥59 years (P = 0.034). (B) Overall survival in non-smoking ESCC patients (P = 0.047). (C) Overall survival in female ESCC patients (P = 0.025). (TIF) [file pone.0103021.s002.tif]

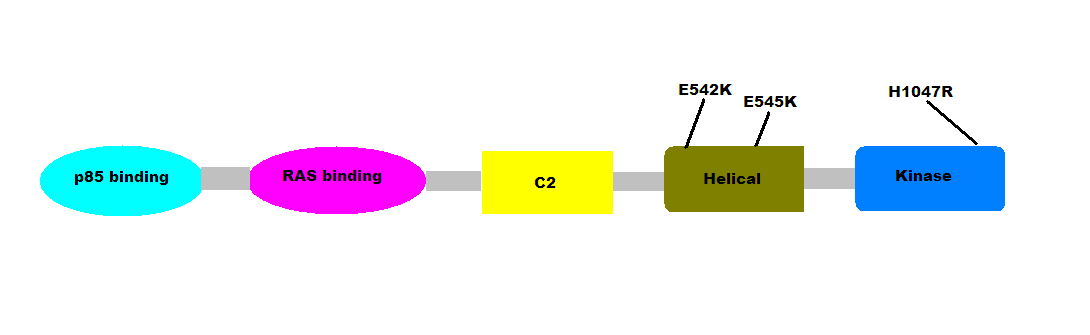

Supplement: Figure S3 — Functional region and hotspot mutation region of PIK3CA. (TIF) [file pone.0103021.s003.tif]

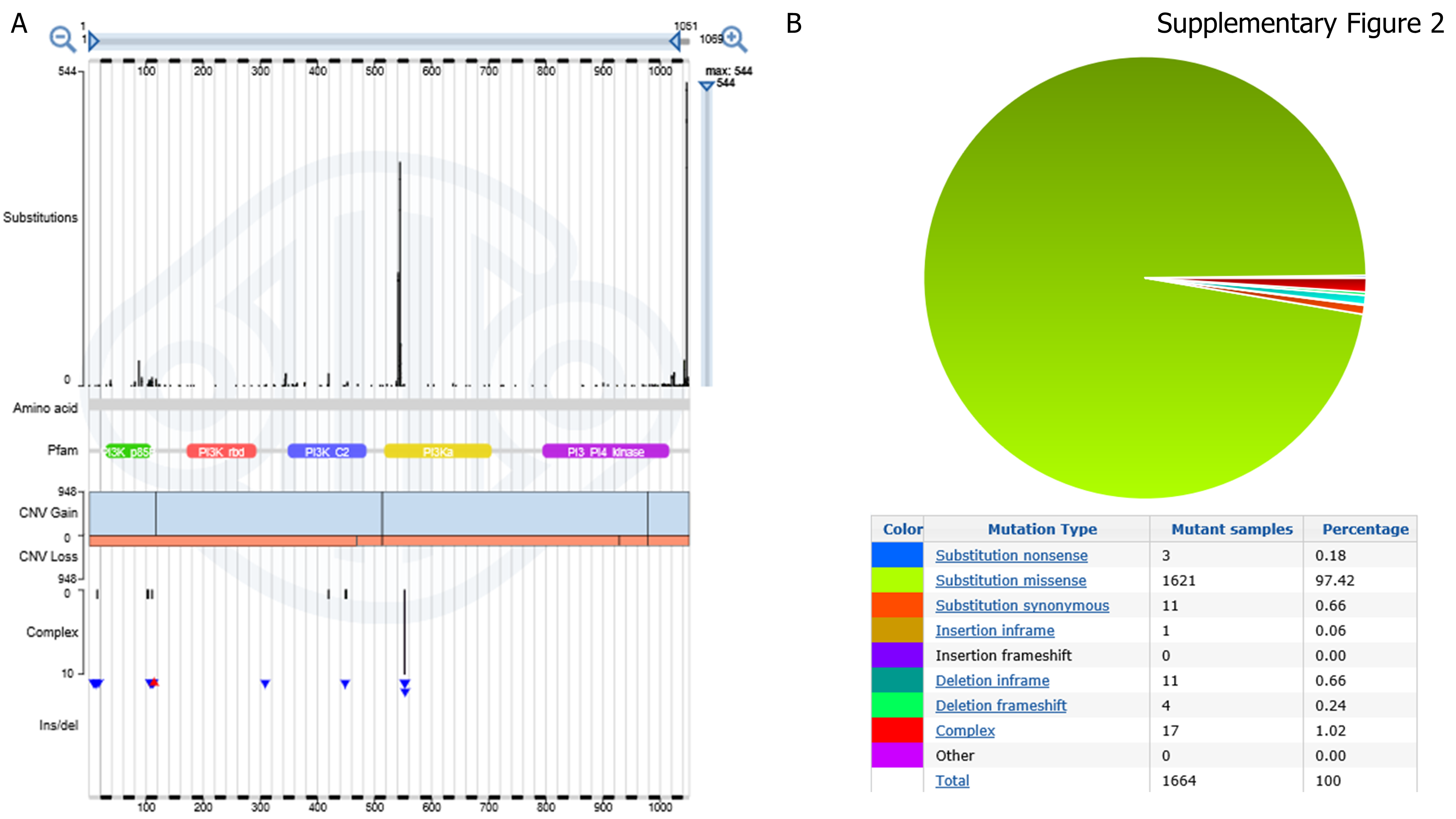

Supplement: Figure S4 — (A) Schematic representation of mutation landscape in PIK3CA as reported in COSMIC database (assessed on May 27, 2014). (B) The overall distribution of known somatic mutations (top panel) and the respective distribution (bottom panel) in tabulated form are provided for cross-reference to somatic landscape in PIK3CA. (TIF) [file pone.0103021.s004.tif]
