# Supplementary material for: PIK3CA Gene Mutations and Overexpression: Implications for Prognostic Biomarker and Therapeutic Target in Chinese Esophageal Squamous Cell Carcinoma
Source: PLoS One. 2014 Jul 23;9(7):e103021. doi: 10.1371/journal.pone.0103021 (PMC4108430; doi:10.1371/journal.pone.0103021)
Supplement: Table S1 — PIK3CA mutations in ESCC. (DOC) [file pone.0103021.s005.doc]

**Table S1**. **PIK3CA mutations in ESCC**

| Exon | Nucleotide | Amino acid | Domain | Cases |
| --- | --- | --- | --- | --- |
| 9 | c.1625A>G | p.E542G | Helical | 3 |
| 9 | c.1634A>C | p.E545A | Helical | 24 |
| 9 | c.1633G>C | p.E545K | Helical | 2 |
| 9 | c.1633G>A | p.E545Q | Helical | 1 |
