# Supplementary material for: PIK3CA Gene Mutations and Overexpression: Implications for Prognostic Biomarker and Therapeutic Target in Chinese Esophageal Squamous Cell Carcinoma
Source: PLoS One. 2014 Jul 23;9(7):e103021. doi: 10.1371/journal.pone.0103021 (PMC4108430; doi:10.1371/journal.pone.0103021)
Supplement: Table S2 — PIK3CA expression in ESCC and normal tissues. (DOC) [file pone.0103021.s006.doc]

**Table S2.** PIK3CA expression in ESCC and normal tissues

| **Groups** | **N** | **PIK3CA** | | **P value** |
| --- | --- | --- | --- | --- |
|  |  | **Positive(+)(%)** | **Negative(-)(%)** |  |
| **Carcinoma** | 406 | 250(61.6) | 156(38.4) | <0.001^a^ |
| **Normal tissue** | 223 | 41(18.4) | 182(81.6) |  |

^a^The *P*-value is significant.
