# Supplementary material for: PIK3CA Gene Mutations and Overexpression: Implications for Prognostic Biomarker and Therapeutic Target in Chinese Esophageal Squamous Cell Carcinoma
Source: PLoS One. 2014 Jul 23;9(7):e103021. doi: 10.1371/journal.pone.0103021 (PMC4108430; doi:10.1371/journal.pone.0103021)
Supplement: Table S3 — PIK3CA expression and PIK3CA mutations in the ESCC patients. (DOC) [file pone.0103021.s007.doc]

**Table S3. PIK3CA expression and PIK3CA mutations in the ESCC patients**

|  | | **PIK3CA** | | **r** | **P value** |
| --- | --- | --- | --- | --- | --- |
|  |  | **Positive(+)** | **Negative(-)** |  |  |
| **PIK3CA** | **Mutant** | 21 | 9 | 0.049 | 0.326 |
|  | **Wild-type** | 229 | 147 |  |  |
